# Prognosis and Prophylactic Regional Nodal Irradiation in Breast Cancer Patients With the First Isolated Chest Wall Recurrence After Mastectomy

**DOI:** 10.3389/fonc.2020.600525

**Published:** 2021-02-10

**Authors:** Xu-Ran Zhao, Liang Xuan, Jun Yin, Yu Tang, Hui-Ru Sun, Hao Jing, Yong-Wen Song, Jing Jin, Yue-Ping Liu, Hui Fang, Hua Ren, Bo Chen, Yuan Tang, Ning Li, Shu-Nan Qi, Ning-Ning Lu, Yong Yang, Ye-Xiong Li, Bing Sun, Shi-Kai Wu, Shu-Lian Wang

**Affiliations:** ^1^ Department of Radiation Oncology, National Cancer Center/National Clinical Research Center for Cancer/Cancer Hospital, Chinese Academy of Medical Sciences and Peking Union Medical College, Beijing, China; ^2^ Department of Radiation Oncology, The Fifth Medical Center, Chinese People's Liberation Army (PLA) General Hospital, Beijing, China; ^3^ Department of Medical Oncology, Peking University First Hospital, Beijing, China

**Keywords:** breast neoplasm, chest wall recurrence, regional failure patterns, radiotherapy, regional nodal irradiation

## Abstract

**Background and Purpose:**

Optimal radiation target volumes for breast cancer patients with their first isolated chest wall recurrence (ICWR) after mastectomy are controversial. We aimed to analyze the regional failure patterns and to investigate the role of prophylactic regional nodal irradiation (RNI) for ICWR.

**Materials and Methods:**

Altogether 205 patients with ICWR after mastectomy were retrospectively analyzed. Post-recurrence progression-free survival (PFS) and overall survival (OS) rates were calculated by Kaplan-Meier method and the differences were compared with Log-rank test. Competing risk model was used to estimate the subsequent regional recurrence (sRR) and locoregional recurrence (sLRR) rates, and the differences were compared with Gray test.

**Results:**

The 5-year sRR rate was 25.2% with median follow-up of 88.6 months. Of the 52 patients with sRR, 30 (57.7%) recurred in the axilla, 29 (55.8%) in supraclavicular fossa (SC), and five (9.6%) in internal mammary nodes. Surgery plus radiotherapy was independently associated with better sLRR and PFS rates (p<0.001). The ICWR interval of ≤ 4 years was associated with unfavorable sRR (p=0.062), sLRR (p=0.014), PFS (p=0.001), and OS (p=0.005). Among the 157 patients who received radiotherapy after ICWR, chest wall plus RNI significantly improved PFS (p=0.004) and OS (p=0.021) compared with chest wall irradiation alone. In the 166 patients whose ICWR interval was ≤ 4 years, chest wall plus RNI provided the best PFS (p<0.001) and OS (p=0.022) compared with chest wall irradiation alone or no radiotherapy.

**Conclusion:**

Patients with ICWR have a high-risk of sRR in SC and axilla. Chest wall plus RNI is recommended.

## Introduction

Breast cancer is a common malignancy in women worldwide, and mastectomy is one important surgical procedure. With multimodality management, approximately 5 to 30% of breast cancer patients recurred at locoregional sites after mastectomy. Among them, 2/3 developed an isolated locoregional recurrence (LRR) without concomitant distant metastasis (DM) (1–4). The chest wall is a frequent site for isolated LRR. Several previous studies have demonstrated that the prognosis of isolated chest wall recurrence (ICWR) is better than isolated LRR involving regional lymph nodes, and a substantial proportion of patients with ICWR can enjoy a long-term survival after curative therapy (5–8).

Patients with ICWR are often treated with multimodality approaches, including excision of the recurrent tumor, radiotherapy, and systemic therapy. However, controversy exists as to the optimal radiation target volumes for isolated LRR, with most advocating irradiation of all local and regional areas (9), whereas others recommending elective irradiation of the chest wall and selected nodal regions (5, 10), or involved field radiotherapy only (11). The value of prophylactic regional nodal irradiation (RNI) for patients with ICWR has not been fully assessed, and the results have been hampered either by the research population or by the time period studied. Further, modern systemic therapy has not only decreased the risk of DM, but has also decreased the risk of LRR, which has raised the question concerning the value of RNI in the contemporary era.

The present retrospective study aimed to assess the prognosis and the incidence and patterns of subsequent locoregional recurrence in breast cancer patients with ICWR, and to evaluate the role of prophylactic RNI.

## Materials and Methods

**Figure 1:**
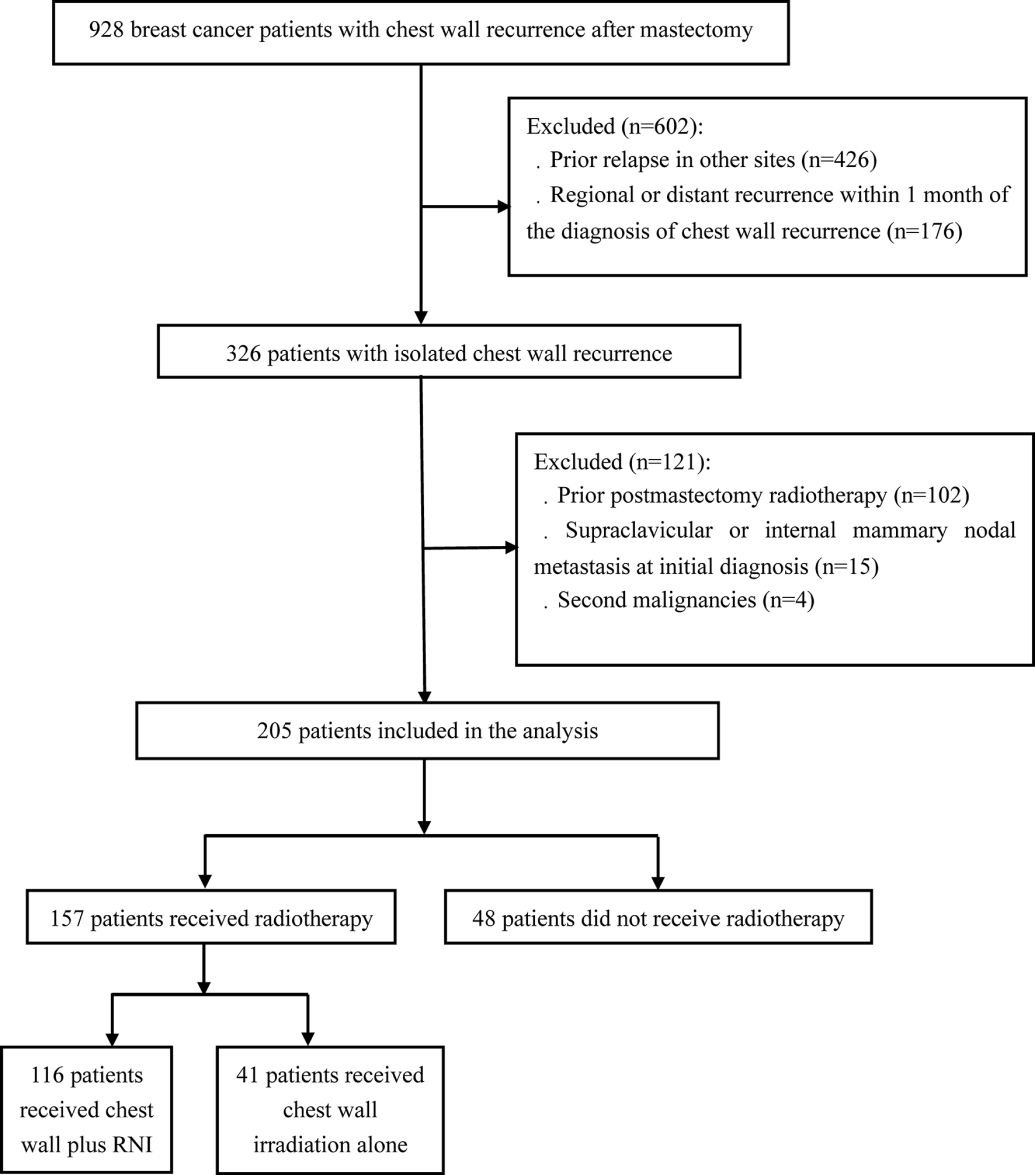
Flow diagram of breast cancer patients included in the study.

A total of 928 breast cancer patients with chest wall recurrence following mastectomy were treated at the National Cancer Center and Chinese PLA General Hospital from October 1998 to April 2018. ICWR was defined as any relapse within the ipsilateral chest wall without prior or concomitant relapse in other sites, and all recurrences were confirmed by pathologic or radiographic evidence. Upon review of the patients we identified, 205 eligible patients with ICWR met the following criteria: no prior relapse in other sites, no regional (axillary, supraclavicular, or internal mammary lymph node) recurrence or DM within 1 month of chest wall recurrence, no postmastectomy radiotherapy, no supraclavicular or internal mammary nodal metastasis at initial diagnosis, and no second malignancies (**Figure 1**). The complete medical records of eligible patients were reviewed, and follow-up data were obtained from hospital records or from correspondence directly with the patient or their family. Computed tomography and nodal ultrasound were routinely used for follow-up. The present study was approved by the Institutional Review Board of Cancer Hospital, Chinese Academy of Medical Sciences (approval number 15-057/984) and the Institutional Review Board of the Fifth Medical Center, Chinese PLA General Hospital (approval number ky-2020-5-8).

The ICWR interval was defined as the time from mastectomy to the date of diagnosis of ICWR. The endpoints included subsequent regional recurrence (sRR), subsequent locoregional recurrence (sLRR), progression-free survival (PFS), and overall survival (OS). sRR was defined as any recurrence within the ipsilateral axillary, supraclavicular fossa (SC), or internal mammary nodes (IMN) after salvage treatment for ICWR. sLRR was defined as the disease progression within the chest wall and/or sRR. PFS event was defined as sLRR, DM, or death attributed to any cause. OS event was defined as death attributed to any cause. Time to survival and/or failure was calculated from the date of diagnosis of ICWR.

The Kaplan-Meier method was used to calculate the survival rates, and the differences were compared using the Log-rank test. The competing risk model was used to estimate the sRR, sLRR, and DM rates, and the differences were compared using the Gray test. Competing risk events for sRR, sLRR, and DM were death without sRR, death without sLRR and death without DM, respectively. Multivariate analysis was performed using Cox logistic and Fine–Gray regression. In addition, we used the Maxstat method to identify the optimal cut-off value of ICWR interval for outcomes (12). The characteristics of the subjects were compared using the Fisher exact or χ2 test. Statistical analyses were performed using cmprsk (https://cran.r-project.org/web/packages/cmprsk/) and Maxstat (https://cran.r-project.org/web/packages/maxstat/index.html) package in R v3.6.0 (http://www.r-project.org/) and SPSS Statistics v24.0 (IBM Corp., Armonk, NY, USA). All P values were two-sided, and a value of less than 0.05 was considered to be significant.

## Results

### Patient Characteristics

Of the 205 patients, 200 (97.6%) patients were pathologically confirmed with ICWR from surgical specimens (n=151) or fine needle aspirations (n=49), and five (2.4%) were diagnosed clinically. The median age at the initial diagnosis of breast cancer was 47 years old (range of 20–90 years old). All patients received a mastectomy. Axillary lymph node dissection was performed in 203 (99.0%) patients, and two (1.0%) patients with pN0 disease underwent sentinel node biopsy alone. The median number of nodes examined was 15 (range of 3–40). The patient, tumor, and treatment characteristics are summarized in **Table 1**. The median interval from mastectomy to ICWR was 20.9 months (range of 1.5–152.7 months). The median size of the ICWR was 1.5 cm (range of 0.3–20.0 cm). After ICWR, 147 (71.7%) patients received chemotherapy, 97 (47.3%) received endocrine therapy, and nine (4.4%) received anti-HER2 targeted therapy. A total of 151 (73.7%) patients received surgery; among them, 82 (54.3%) had R0, three (1.9%) R1, six (4.0%) R2, 59 (39.1%) Rx chest wall tumor resection alone, and one (0.7%) had Rx chest wall tumor resection plus axillary lymph node dissection. A total of 157 (76.6%) patients received irradiation to the chest wall ± regional nodes with conventional fractionation. CT-based radiotherapy technique has not been used until 2016, and most patients were treated with two-dimensional radiotherapy technique. A large field encompassing the entire chest wall was used in all patients to deliver a median total dose of 50 Gy (range of 10–73.5 Gy). A local “boost” therapy was used in 80 (51.0%) patients, and the median total dose to recurrent tumor or tumor bed after resection was 65 Gy (range of 50–76 Gy). Bolus was routinely used for at least 60% course of radiotherapy to the chest wall. Among the 116 (73.9%) patients who received RNI, 115 (99.1%) were given SC irradiation, 16 (13.8%) received axillary irradiation, and five (4.3%) received IMN irradiation. The nodal sites were treated to a median dose of 50 Gy (range of 20–64 Gy).

**Table 1:** Patient, tumor, and treatment characteristics of 205 breast cancer patients with ICWR.

Characteristics	No. of Patients (%)
Age at initial diagnosis (years)	
≤50	134 (65.4)
>50	71 (34.6)
Initial Location	
Inner/central quadrant	49 (23.9)
Other quadrants	99 (48.3)
Unknown	57 (27.8)
Initial staging*	
I–II	161 (78.5)
III	37 (18.0)
Unknown	7 (3.4)
Initial histological grade	
I–II	77 (37.6)
III	33 (16.1)
Unknown	95 (46.3)
Initial chemotherapy	
Yes	190 (92.7)
No	15 (7.3)
Initial endocrine therapy^†^	
Yes	100 (75.2)
No	32 (24.1)
Unknown	1 (0.8)
Initial anti-HER2 target therapy^‡^	
Yes	2 (5.6)
No	33 (91.7)
Unknown	1 (2.8)
ICWR interval (years)	
≤4	166 (80.9)
>4	39 (19.1)
No. of ICWRs	
1	169 (82.4)
2	11 (5.4)
≥3	20 (9.8)
Unknown	5 (2.4)
Molecular subtype#	
Luminal-HER2 negative	100 (48.8)
Luminal-HER2 positive	22 (10.7)
HER2-enriched	16 (7.8)
Triple-negative	48 (23.4)
Unknown	19 (9.3)
Treatment modalities for ICWR	
Locoregional treatment alone	26 (12.7)
Systemic treatment alone	22 (10.7)
Locoregional + systemic treatment	157 (76.6)
Locoregional treatment for ICWR	
Surgery + radiotherapy	125 (61.0)
Radiotherapy alone	32 (15.6)
Surgery alone	26 (12.7)
None	22 (10.7)

HER2, human epidermal growth factor receptor 2.

*For the 17 patients that received neoadjuvant chemotherapy, we used the stage that was higher (clinical or pathological) to reflect the initial tumor burden; ^†^Only hormone-receptor positive patients were included; ^‡^Only HER2 positive patients were included. # For the 92 patients who had sufficient information for determination of the molecular subtype of ICWR, we used the molecular subtypes of ICWR. For the 94 patients who only had sufficient information for determination of the molecular subtype at initial diagnosis, we used the initial molecular subtypes.

### Outcomes and Failure Patterns of the Entire Cohort

Following a median follow-up of 88.6 months (range of 1.6–220.6 months) after ICWR, there were 160 (78.0%) patients that experienced subsequent recurrence. The results of the first failure were as follows: locoregional in 63 (39.4%) patients, distant in 73 (45.6%) patients, and simultaneous locoregional and distant in 24 (15.0%) patients. A total of 103 (50.2%) patients developed sLRR. The 5-year cumulative sLRR rate was 49.0%, and the median interval from ICWR to sLRR was 12.5 months (range of 1.2–118.7 months). A total of 52 (25.4%) patients developed sRR. The 5-year cumulative sRR rate was 25.2%, and the median interval from ICWR to sRR was 13.8 months (range to 1.9–117.3 months). A total of 138 (67.3%) patients developed DM and 103 (50.2%) patients died. The 5-year cumulative PFS rate was 22.7%, and the median PFS after ICWR was 16.1 months (range of 1.2–117.3 months). The 5-year cumulative OS rate was 53.9%, and the median OS after ICWR was 65.9 months (range of 7.6–162.6 months).

**Figure 2:**
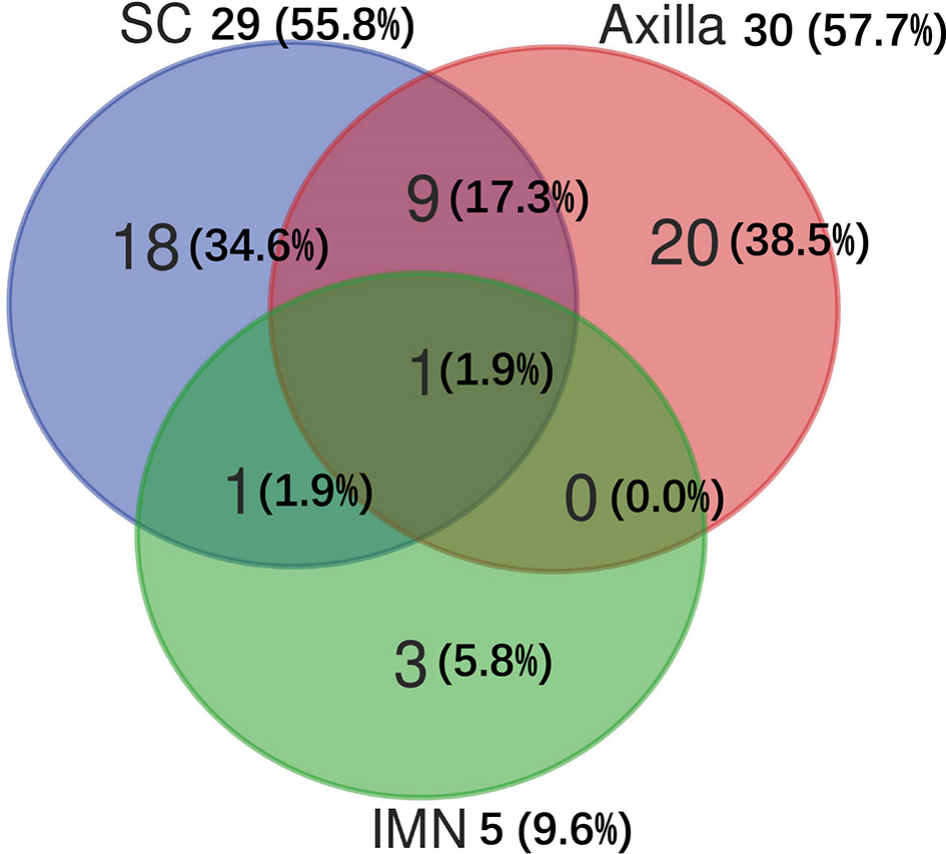
Distribution of subsequent regional recurrences in 52 breast cancer patients. IMN, Internal mammary nodes; SC, Supraclavicular fossa.

Among 103 patients who developed sLRR, 31 (30.1%) had regional node recurrence only, 21 (20.4%) had both regional node and chest wall recurrences, and 51 (49.5%) had chest wall recurrence only. Of the 52 patients with sRR, 30 (57.7%) recurred in the axilla, 29 (55.8%) in the SC, and five (9.6%) in the IMN (**Figure 2**).

**Figure 3:**
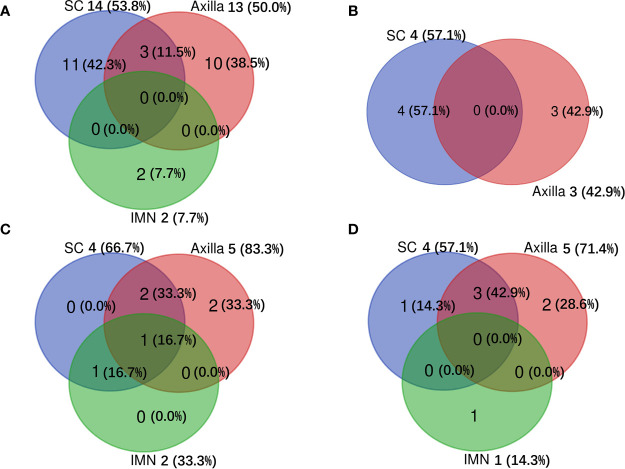
Distribution of subsequent regional recurrences among different molecular subtypes of breast cancer. **(A)** for luminal-HER2 negative, **(B)** for luminal-HER2 positive, **(C)** for HER2-enriched, and **(D)** for triple-negative tumors. IMN, Internal mammary nodes; SC, Supraclavicular fossa.

There were 186 patients who had sufficient information for determination of the molecular subtype of breast cancer (**Table 1**). For sLRR patients with luminal-HER2 negative, luminal-HER2 positive, HER2-enriched, and triple-negative tumors, 16 (31.4%), 6 (54.5%), 2 (20.0%), 3 (15.0%) had regional node recurrence only, 10 (19.6%), 1 (9.1%), 4 (40.0%), 4 (20.0%) had both regional node and chest wall recurrences, and 25 (49.0%), 4 (36.4%), 4 (40.0%), 13 (65.0%) had chest wall recurrence only. For sRR patients with luminal-HER2 negative, luminal-HER2 positive, HER2-enriched, and triple-negative tumors, 13 (50.0%), 3 (42.9%), 5 (83.3%), 5 (71.4%) recurred in the axilla, 14 (53.8%), 4 (57.1%), 4 (66.7%), 4 (57.1%) in the SC, and 2 (7.7%), 0 (0.0%), 2 (33.3%), 1 (14.3) in the IMN (**Figure 3**).

### The Role of Locoregional Treatment for ICWR of the Entire Cohort

**Figure 4:**
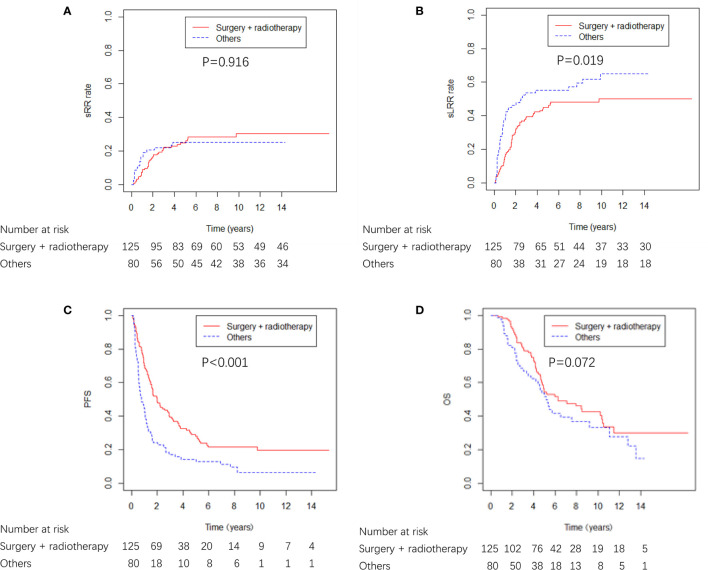
sRR, sLRR, PFS, and OS curves from locoregional treatment given to breast cancer patients with ICWR.

A total of 125 (61.0%) patients received surgery plus radiotherapy, and 80 (39.0%) patients received either surgery or radiotherapy alone, or no locoregional therapy (**Table 1**). The characteristics were well balanced between the four groups (surgery + radiotherapy, surgery alone, radiotherapy alone and none) (**Supplementary Table 1**). Surgery plus radiotherapy was associated with better sLRR and PFS compared with other therapies, and there was a nonsignificant trend toward improved OS with surgery plus radiotherapy compared with other therapies, and there was no difference in sRR between the two groups (**Figure 4**).

Maxstat analysis showed that as an sRR prognostic factor, the optimal cut-off value of ICWR interval was 4.0 years. The univariate analysis showed that an ICWR interval of ≤ 4 years was an unfavorable prognostic factor for sRR, sLRR, PFS, and OS (**Supplementary Table 2**). The multivariate analysis included the most relevant prognostic variables identified from univariate analysis (initial age, initial staging, molecular subtype, ICWR interval ≤ 4 years, locoregional treatment and treatment modalities for ICWR). Surgery plus radiotherapy was an independent favorable prognostic factor for PFS. Locoregional plus systemic treatment was independent favorable prognostic factors for both PFS and OS. ICWR interval of > 4 years was an independent favorable prognostic factor for sRR, sLRR, PFS, and OS. Initial stage and molecular subtype were independent prognostic factors for OS (**Table 2**).

**Table 2:** Multivariate analysis of sRR, sLRR, PFS, and OS in 205 breast cancer patients with ICWR.

Variable	sRR		sLRR		PFS		OS	
	HR (95% CI)	P	HR (95% CI)	P	HR (95% CI)	P	HR (95% CI)	P
Age at initial diagnosis		0.500		0.900		0.455		0.180
≤50 years	1.00		1.00		1.00		1.00	
>50 years	1.23 (0.67–2.25)		0.97 (0.61–1.54)		0.87 (0.61–1.25)		1.34 (0.87–2.06)	
Initial stage		0.210		0.910		0.235		0.021
I–II	1.00		1.00		1.00		1.00	
III	0.55 (0.21–1.42)		1.03 (0.58–1.85)		1.30 (0.84–2.02)		1.86 (1.10–3.14)	
Molecular subtype#		0.230		0.360		0.283		0.002
Luminal-HER2 negative	1.00		1.00		1.00		1.00	
Luminal-HER2 positive	1.17 (0.53–2.57)		0.74 (0.39–1.40)		1.19 (0.71–1.99)		1.49 (0.80–2.79)	
HER2-enriched	1.49 (0.57–3.86)		1.16 (0.56–2.41)		1.36 (0.77–2.41)		3.36 (1.69–6.67)	
Triple-negative	0.51 (0.21–1.23)		0.72 (0.40–1.29)		0.78 (0.50–1.20)		0.86 (0.51–1.44)	
ICWR interval		0.034		0.008		0.003		0.012
>4 years	1.00		1.00		1.00		1.00	
≤4 years	2.94 (1.08–7.99)		2.31 (1.25–4.27)		1.99 (1.26–3.15)		2.25 (1.20–4.24)	
Locoregional treatment for recurrence		0.540		0.150		0.001		0.279
Surgery + radiotherapy	1.00		1.00		1.00		1.00	
Others	0.81 (0.42–1.56)		1.39 (0.88–2.18)		1.89 (1.32–2.70)		1.27 (0.82–1.97)	
Treatment modalities for recurrence		0.680		0.300		0.004		<0.001
Locoregional + systemic treatment	1.00		1.00		1.00		1.00	
Locoregional or systemic treatment alone	1.18 (0.54–2.55)		1.33 (0.78–2.29)		1.86 (1.22–2.81)		2.73 (1.69–4.39)	

HER2, human epidermal growth factor receptor 2.

### Effect of Regional Nodal Irradiation on the Prognosis

**Figure 5:**
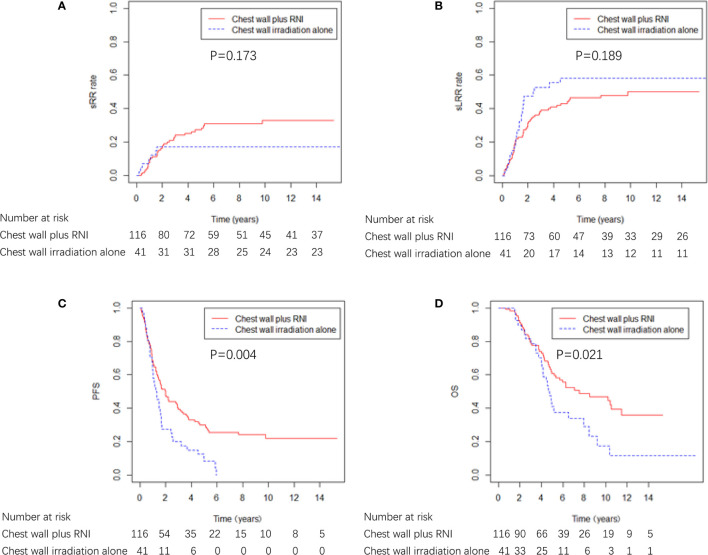
sRR, sLRR, PFS, and OS curves by radiation volume in 157 breast cancer patients that received radiotherapy.

Among the 157 patients who received radiotherapy after ICWR, 41 (26.1%) patients received chest wall irradiation alone, and 116 (73.9%) patients received chest wall plus RNI. The characteristics of the two groups are shown in **Supplementary Table 3**. The characteristics were well balanced between the two groups; however, the chest wall plus RNI group had more patients who were >50 years old (p = 0.020) and had ≥ 2 sites of ICWR (p = 0.017). The chest wall plus RNI group showed significantly better PFS and OS than the chest wall irradiation alone group, and there were no differences in sRR or sLRR between the two groups (**Figure 5**). The chest wall plus RNI group showed significantly lower DM than that of the chest wall irradiation alone group (59.0% vs. 79.2% at 5 years, p = 0.003).

**Figure 6:**
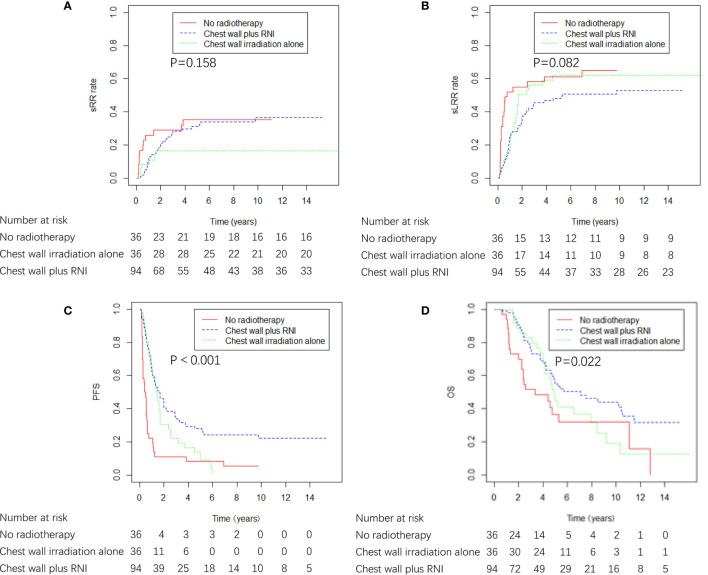
sRR, sLRR, PFS, and OS curves by radiation volume in 166 breast cancer patients whose ICWR interval was ≤ 4 years.

The ICWR interval of ≤ 4 years was the variable most strongly predictive of adverse sRR, and the 5-year sRR rate was 28.8% and 9.0% for patients whose ICWR interval of ≤ 4 years and >4 years (p = 0.019), therefore, the influence of RNI was evaluated in 166 patients whose ICWR interval was ≤ 4 years. Chest wall plus RNI (n=94) provided the best OS and PFS compared with patients who received chest wall irradiation alone (n=36) or no radiotherapy (n=36). There was a nonsignificant trend toward reduced sLRR with chest wall plus RNI compared with chest wall irradiation alone or no radiotherapy, but no differences in sRR were detected among the three groups (**Figure 6**). Chest wall plus RNI significantly reduced the risk of DM as compared with chest wall irradiation alone or no radiotherapy (59.1 vs. 77.8 vs. 75.0% at 5 years, p = 0.004).

## Discussion

In the present study, we found that the sLRR risk was high in patients with ICWR, which indicates the indispensability of locoregional treatment, including both surgery and radiotherapy. Surgical excision is generally the preferred initial treatment. Thus, not only the tumor burden is reduced, but also histological and immunohistochemical diagnosis of the recurrence can be established to help determine systemic-treatment decisions. Early reports have revealed that excision alone results in sLRR rates of 60–75% (13, 14), which indicates the need for adjuvant radiotherapy. In previous studies, surgery plus radiotherapy has been demonstrated to achieve better survival outcome than either surgery or radiotherapy alone (8, 14, 15). Our results showed that surgery plus radiotherapy was an independent favorable prognostic factor for PFS but not for OS in the multivariate analysis. The failure of surgery plus radiotherapy to improve OS could be attributable to the insufficient number of patients analyzed in our study, or the improvements in the effectiveness of systemic therapy as salvage therapy for the subsequent recurrence after the ICWR. Since there was a significant improved PFS (HR 1.89, 95%CI 1.32–2.70; p = 0.001) and a nonsignificant trend toward improved OS with surgery plus radiotherapy (HR 1.27, 95%CI 0.82–1.97; p = 0.279), comprehensive locoregional treatment, including both surgery and radiotherapy, was recommended for these patients.

Previous reports have shown that sLRR not only reduced the quality of life, but also portended an unfavorable survival outcome (5, 16). Our results showed that 50.2% of patients developed sLRR, among which sRR accounted for 50.5%. Multivariate analysis demonstrated that ICWR interval was an important non-treatment-related prognostic factor for sRR (HR 2.9), sLRR (HR 2.3), PFS (HR 2.0) and OS (HR 2.3), which indicated the discrepancy in biological aggressiveness between patients whose ICWR interval was >4 years and those with earlier recurrence. Previous studies have shown that the survival following locoregional and systemic therapies for isolated LRR might be adversely affected by short interval from mastectomy to recurrence (6, 17), some showed that the disease-free interval of less than 1 year was significantly associated with worse OS (18, 19), while most studies observed the disease-free interval of less than 2 years was significantly associated with worse locoregional control (14), distant metastasis-free survival (20, 21), PFS (15, 21) or OS (14, 15, 19–23). However, the interval they used as a cut-off was generally defined according to their experience. To the best of our knowledge, our study is the first one to use sRR as a primary endpoint in order to evaluate the effect of RNI on the prognosis for patients with ICWR. In addition, we used the Maxstat method to identify the optimal cut-off value of ICWR interval for outcomes, which is more reasonable. Early isolated LRR events represent biologically aggressive disease, whereas late recurrences indicate indolent disease (24). Thus, more effective local treatment, such as chest wall plus RNI, may be warranted in patients with early isolated LRR. Only a few previous studies have analyzed prognostic factors for sLRR and sRR by multivariate analysis, and showed that ER status of the recurrent tumor (14, 21, 25), lymphovascular invasion of the recurrent tumor (25), and initial staging (24) were associated with sLRR. In addition, ER status along with lymphovascular invasion of the recurrent tumor (25) were associated with sRR. The differences between the previous reports and the present study is explained by the type of patient population. Most previous studies have included patients with various LRR patterns rather than only ICWR (14, 21, 24), and some studies have only analyzed patients with ipsilateral breast tumor recurrence after breast-conserving surgery (25). Furthermore, the use of a more effective systemic therapy in our study possibly effected the prognosis.

There was no consistent plan with regard to irradiation volume for either inclusion or exclusion of uninvolved regions in patients with ICWR. Our results showed that chest wall plus RNI significantly improved PFS and OS compared no RNI in patients who received radiotherapy and in patients whose ICWR interval was ≤ 4 years, which indicates the necessity for RNI in these patients. The effect of RNI on survival may be due to decreased risk of both sLRR and DM by irradiation of subclinical disease in regional sites, thus preventing the dissemination of neoplastic cells. A recently published study revealed that 85.8% of patients with *de novo* metastatic breast cancer harbor regional lymph node disease at presentation (26), which is consistent with the hypothesis that regional involvement may precede metastatic dissemination. That is a possible explanation for our results and the previous findings that RNI reduces distant recurrences (27–30). The value of prophylactic RNI has been a topic of debate; some studies have suggested that the risk of developing subsequent failures in uninvolved sites is low after involved-field radiotherapy only encompassing the recurrence sites (14, 19). However, Chen et al. reported that the percentage of sRR was 20% in patients with ICWR after chest wall irradiation alone (5). Toonkel et al. reported a survival advantage with prophylactic RNI (9), but Deutsch et al. reported a better 5-year OS for patients receiving chest wall irradiation alone for ICWR compared with patients receiving chest wall plus RNI (11). Similar results were observed by Willner et al., whose findings showed a significant survival advantage with recurrence-site irradiation alone over total locoregional irradiation for patients with isolated LRR (18). Two previous studies showed that the second recurrence rate in the SC was reduced in patients that received prophylactic SC irradiation (10, 13). Patients treated with radiotherapy to a limited target volume probably had less tumor burden than those treated more extensively (11, 18), thus the advantage of recurrence-site irradiation alone should be interpreted with reservation. Most previous studies included patients with various LRR patterns and some did not exclude patients with initial post-mastectomy radiotherapy. Moreover, the sample size was quite small and the follow-up time maybe not long enough to observe the distinction between different patient groups. Systemic treatments as adjuvant or salvage therapies varied considerably due to the different time period studied. Whereas our study included a large number of patients with pure ICWR, mainly treated with modern systemic therapies, which have decreased the incidence of subsequent distant dissemination and made it possible for enlarging irradiation volume to provide superior outcomes.

In our cohort, 56.6% of the patients had received prophylactic RNI, and the 5-year sRR rate was 25.2%. The studies on the LRR patterns after ICWR were scarce. The failure patterns were associated with both disease status and upfront treatment. In the studies evaluating the patterns of nodal involvement for patients with *de novo* metastatic breast cancer, or the first LRR after adjuvant radiotherapy in patients who had undergone breast conservation surgery or mastectomy, axilla was the most common involved site, followed by SC and IMN (26, 31). In contrast, we found that axilla and SC were the most common sites of sRR after ICWR. Notably, among 116 patients who received RNI in our study, 99.1% included SC, whereas only 13.8% included axilla. The high-risk of sRR may be attributed to inadequate regional target volume. Additionally, most patients in the present study were treated with traditional two-dimensional radiotherapy techniques, such as a single anterior field or opposed anterior-posterior/posterior-anterior fields to SC and/or axilla, which may result in an inadequate dose delivered to these regions. Previous dosimetric evaluations have shown that conventional radiotherapy techniques exhibited inferior target volume coverage compared with intensity-modulated radiation therapy (32, 33). Additionally, a high sRR rate may be due to radiation-resistant subclinical disease existing in regional sites. A previous study from MD Anderson Cancer Center reported that radiation dose escalation to at least 66 Gy was not sufficient to achieve a detectable improvement in locoregional control rates among patients with LRR, which suggests the intrinsic radiation resistance of the recurrent disease (34). Prior reports observed the relatively low frequency of relapse in the axilla and increased incidence of arm edema caused by axillary irradiation, which concluded that the axilla should not be routinely included in the treatment volume (10, 13). Thus, mapping the anatomic location of sRR in axilla and SC deserves additional study, and the value of RNI should be examined in a prospective randomized trial using modern radiotherapy technique based on CT imaging.

The limitations of our study should be acknowledged. Because this study was retrospective and spanned a long period of time, systemic treatment, such as adjuvant or salvage therapy, varied considerably and confounding factors likely were present in this series. There was a possible underestimate of sLRR because of the limitations of the follow up images and the retrospective nature of this study. Despite these limitations, our study included a large number of patients with ICWR treated in two institutions, and the follow-up time was lengthy. To the best of our knowledge, the present study is the first to identify the prognostic factors and failure patterns of sRR in patients with ICWR, and it provides a direction for prospective studies to improve the treatment of these patients.

## Conclusions

ICWR after mastectomy poses a challenge for clinicians; comprehensive locoregional treatment, including both surgery and radiotherapy, provide the best outcomes for patients with ICWR. The high-risk of sRR in the SC and axilla indicates the possible important role of prophylactic RNI.

## Data Availability Statement

The data analyzed in this study is subject to the following licenses/restrictions: The datasets for this article are not publicly available because of ethical restrictions placed on patient data. Requests to access these datasets should be directed to S-LW, wsl20040118@yahoo.com.

## Ethics Statement

The studies involving human participants were reviewed and approved by The Institutional Review Board of Cancer Hospital, Chinese Academy of Medical Sciences and the Institutional Review Board of the Fifth Medical Center, Chinese PLA General Hospital. Written informed consent for participation was not required for this study in accordance with the national legislation and the institutional requirements.

## Author Contributions

X-RZ and LX: Formal analysis, investigation, data collection, methodology, and writing original draft. JY and H-RS: Investigation, data collection, review original draft and editing. YuT: Data collection, patients care, review original draft and editing. HJ, Y-WS, JJ, Y-PL, HF, HR, BC, YuaT, NL, S-NQ, N-NL, and YY: Patients care, review original draft and editing. Y-XL: Statistics guidance, project administration, patients care, review original draft and editing. BS: Formal analysis and data collection, validation, statistics guidance, patients care, and writing original draft and editing. S-KW: Formal analysis and data collection, validation, statistics guidance, patients care, and writing original draft and editing. S-LW: Formal analysis and data collection, validation, statistics guidance, project administration, patients care, and writing original draft and editing. All authors contributed to the article and approved the submitted version.

## Funding

This work was supported by National Key Projects of Research and Development of China (grant number 2016YFC0904600), Beijing Marathon of Hope, Cancer Foundation of China (grant number LC2016A09), National Natural Science Foundation of China (grant number 81972860).

## Conflict of Interest

The authors declare that the research was conducted in the absence of any commercial or financial relationships that could be construed as a potential conflict of interest.
