# Supplementary material for: Prognosis and Prophylactic Regional Nodal Irradiation in Breast Cancer Patients With the First Isolated Chest Wall Recurrence After Mastectomy
Source: Front Oncol. 2021 Feb 10;10:600525. doi: 10.3389/fonc.2020.600525 (PMC7902693; doi:10.3389/fonc.2020.600525)
Supplement: Supplementary file 1 [file DataSheet_1.docx]

**Supplementary Table 1. General characteristics and treatment-related factors of the four locoregional treatment groups in the entire cohort.**

|  | Surgery + radiotherapy | Surgery alone | Radiotherapy alone | None | P |
| --- | --- | --- | --- | --- | --- |
| Characteristics | No. of Patients　(%) | No. of Patients　(%) | No. of Patients　(%) | No. of Patients　(%) |  |
| Age at initial diagnosis (years) |  |  |  |  | 0.577 |
| ≤ 50 | 83 (66.4) | 19 (73.1) | 20 (62.5) | 12 (54.5) |  |
| >50 | 42 (33.6) | 7 (26.9) | 12 (37.5) | 10 (45.5) |  |
| Initial Location |  |  |  |  | 0.573 |
| Inner/central quadrant | 28 (30.4) | 5 (27.8) | 10 (43.5) | 6 (40.0) |  |
| Other quadrants | 64 (69.6) | 13 (72.2) | 13 (56.5) | 9 (60.0) |  |
| Initial staging* |  |  |  |  | 0.284 |
| I-II | 103 (84.4) | 17 (73.9) | 23 (71.9) | 18 (85.7) |  |
| III | 19 (15.6) | 6 (26.1) | 9 (28.1) | 3 (14.3) |  |
| Initial histological grade |  |  |  |  | 0.649 |
| I-II | 52 (73.2) | 7 (63.6) | 11 (68.8) | 7 (58.3) |  |
| III | 19 (26.8) | 4 (36.4) | 5 (31.3) | 5 (41.7) |  |
| Initial chemotherapy |  |  |  |  | 0.332 |
| Yes | 118 (94.4) | 22 (84.6) | 30 (93.8) | 20 (90.9) |  |
| No | 7 (5.6) | 4 (15.4) | 2 (6.3) | 2 (9.1) |  |
| Initial endocrine therapy |  |  |  |  | 0.644 |
| Yes | 67 (54.0) | 15 (57.7) | 14 (43.8) | 13 (59.1) |  |
| No | 57 (46.0) | 11 (42.3) | 18 (56.3) | 5 (40.9) |  |
| Initial anti-HER2 target therapy |  |  |  |  | 0.111 |
| Yes | 0 (0.0) | 0 (0.0) | 1 (3.1) | 1 (4.5) |  |
| No | 124 (100.0) | 25 (100.0) | 31 (96.9) | 21 (95.5) |  |
| ICWR interval (years) |  |  |  |  | 0.515 |
| ≤ 4 | 102 (81.6) | 20 (76.9) | 28 (87.5) | 16 (72.7) |  |
| > 4 | 23 (18.4) | 6 (23.1) | 4 (12.5) | 6 (27.3) |  |
| No. of ICWR |  |  |  |  | 0.213 |
| 1 | 105 (86.1) | 22 (88.0) | 23 (71.9) | 19 (90.5) |  |
| ≥2 | 17 (13.9) | 3 (12.0) | 9 (28.1) | 2 (9.5) |  |
| Molecular subtype# |  |  |  |  | 0.169 |
| Luminal-HER2 negative | 65 (56.5) | 16 (72.7) | 12 (44.4) | 7 (31.8) |  |
| Luminal-HER2 positive | 10 (8.7) | 2 (9.1) | 6 (22.2) | 4 (18.2) |  |
| HER2-enriched | 10 (8.7) | 1 (4.5) | 3 (11.1) | 2 (9.1) |  |
| Triple-negative | 30 (26.1) | 3 (13.6) | 6 (22.2) | 9 (40.9) |  |
| Chemotherapy for ICWR |  |  |  |  | 0.053 |
| Yes | 84 (67.2) | 18 (69.2) | 24 (75.0) | 21 (95.5) |  |
| No | 41 (32.8) | 8 (30.8) | 8 (25.0) | 1 (4.5) |  |
| Endocrine therapy for ICWR |  |  |  |  | 0.379 |
| Yes | 64 (51.2) | 13 (50.0) | 12 (37.5) | 8 (36.4) |  |
| No | 61 (48.8) | 13 (50.0) | 20 (62.5) | 14 (63.6) |  |
| Anti-HER2 target therapy for ICWR |  |  |  |  | 0.418 |
| Yes | 4 (3.2) | 1 (3.8) | 2 (6.3) | 2 (9.1) |  |
| No | 121 (96.8) | 25 (96.2) | 30 (93.8) | 20 (90.9) |  |
| Systemic treatment for ICWR |  |  |  |  | 0.253 |
| Yes | 107 (85.6) | 22 (84.6) | 28 (87.5) | 22 (100.0) |  |
| No | 18 (14.4) | 4 (15.4) | 4 (12.5) | 0 (0.0) |  |

*Abbreviations:* HER2 = human epidermal growth factor receptor 2

*For the 17 patients that received neoadjuvant chemotherapy, we used the stage that was higher (clinical or pathological) to reflect the initial tumor burden. # For the 92 patients who had sufficient information for determination of the molecular subtype of ICWR, we used the molecular subtypes of ICWR. For the 94 patients who only had sufficient information for determination of the molecular subtype at initial diagnosis, we used the initial molecular subtypes.

**Supplementary Table 2. Univariate analysis of patient characteristics and treatment-related factors for sLRR, sRR, PFS and OS.**

| Characteristic | 5-year sRR (%) | P | 5-year sLRR (%) | P | 5-year PFS (%) | P | 5-year OS (%) | P |
| --- | --- | --- | --- | --- | --- | --- | --- | --- |
| Age at initial diagnosis (years) |  | 0.711 |  | 0.485 |  | 0.921 |  | 0.037 |
| ≤50 | 24.8 |  | 47.8 |  | 22.6 |  | 58.3 |  |
| >50 | 26.0 |  | 51.5 |  | 24.2 |  | 45.9 |  |
| Initial Location |  | 0.210 |  | 0.248 |  | 0.141 |  | 0.910 |
| Inner/central quadrant | 17.9 |  | 40.2 |  | 34.6 |  | 54.0 |  |
| Other quadrants | 24.5 |  | 47.1 |  | 24.6 |  | 57.6 |  |
| Initial stage |  | 0.216 |  | 0.883 |  | 0.116 |  | 0.008 |
| I-II | 26.5 |  | 48.3 |  | 24.3 |  | 57.9 |  |
| III | 17.5 |  | 49.2 |  | 16.5 |  | 30.9 |  |
| Initial histological grade |  | 0.446 |  | 0.198 |  | 0.772 |  | 0.631 |
| I-II | 17.6 |  | 49.4 |  | 24.0 |  | 49.9 |  |
| III | 28.6 |  | 38.0 |  | 20.5 |  | 45.3 |  |
| Initial chemotherapy |  | 0.020 |  | 0.596 |  | 0.688 |  | 0.558 |
| Yes | 27.2 |  | 49.2 |  | 22.8 |  | 53.5 |  |
| No | 0 |  | 46.7 |  | 26.7 |  | 59.2 |  |
| Initial endocrine therapy |  | 0.478 |  | 0.295 |  | 0.344 |  | 0.080 |
| Yes | 23.5 |  | 46.1 |  | 25.6 |  | 61.8 |  |
| No | 27.5 |  | 53.0 |  | 20.6 |  | 44.6 |  |
| Initial anti-HER2 target therapy |  | - |  | - |  | - |  | - |
| Yes | - |  | - |  | - |  | - |  |
| No | 25.1 |  | 49.4 |  | 22.8 |  | 53.8 |  |
| ICWR interval |  | 0.018 |  | 0.010 |  | 0.018 |  | 0.015 |
| ≦4 years | 28.9 |  | 54.2 |  | 20.3 |  | 48.6 |  |
| >4 years | 9.0 |  | 26.4 |  | 35.8 |  | 76.9 |  |
| No. of ICWR |  | 0.801 |  | 0.410 |  | 0.471 |  | 0.460 |
| 1 | 26.1 |  | 50.2 |  | 21.0 |  | 52.0 |  |
| 2 | 21.6 |  | 30.0 |  | 36.4 |  | 80.0 |  |
| ≥3 | 20.0 |  | 51.9 |  | 26.2 |  | 61.2 |  |
| Tumor size of ICWR (continuous) |  | 0.370 |  | 0.520 |  | 0.852 |  | 0.337 |
| Molecular subtype# |  | 0.281 |  | 0.786 |  | 0.143 |  | 0.001 |
| Luminal-HER2 negative | 23.0 |  | 45.4 |  | 29.8 |  | 59.8 |  |
| Luminal-HER2 positive | 32.8 |  | 51.5 |  | 5.5 |  | 55.6 |  |
| HER2-enriched | 38.8 |  | 62.5 |  | 6.3 |  | 9.5 |  |
| Triple-negative | 16.4 |  | 46.5 |  | 22.7 |  | 51.5 |  |
| Surgical margin of ICWR |  | 0.103 |  | 0.011 |  | 0.046 |  | 0.278 |
| R0 | 22.1 |  | 40.8 |  | 38.2 |  | 64.1 |  |
| R1/2 | 0 |  | 77.8 |  | 11.1 |  | 22.5 |  |
| Locoregional treatment for ICWR |  | 0.922 |  | 0.019 |  | <0.001 |  | 0.125 |
| Surgery + radiotherapy | 25.1 |  | 45.1 |  | 28.5 |  | 54.2 |  |
| Others (surgery or radiotherapy alone, or none) | 25.3 |  | 55.2 |  | 14.7 |  | 53.6 |  |
| Treatment modalities for ICWR |  | 0.534 |  | 0.036 |  | <0.001 |  | <0.001 |
| Locoregional or systemic treatment alone | 30.0 |  | 62.4 |  | 10.4 |  | 28.9 |  |
| Locoregional + systemic treatment | 23.8 |  | 45.2 |  | 26.9 |  | 61.4 |  |

*Abbreviations:* HER2 = human epidermal growth factor receptor 2

# For the 92 patients who had sufficient information for determination of the molecular subtype of ICWR, we used the molecular subtypes of ICWR. For the 94 patients who only had sufficient information for determination of the molecular subtype at initial diagnosis, we used the initial molecular subtypes.

**Supplementary Table 3. General characteristics and treatment-related factors by radiation volume in 157 breast cancer patients that received radiotherapy.**

|  | Chest wall plus RNI | Chest wall irradiation alone | P |
| --- | --- | --- | --- |
| Characteristics | No. of Patients　(%) | No. of Patients　(%) |  |
| Age at initial diagnosis (years) |  |  | 0.020 |
| ≤ 50 | 70 (60.3) | 33 (80.5) |  |
| >50 | 46 (39.7) | 8 (19.5) |  |
| Initial Location |  |  | 0.666 |
| Inner/central quadrant | 30 (34.1) | 8 (29.6) |  |
| Other quadrants | 58 (65.9) | 19 (70.4) |  |
| Initial stage* |  |  | 0.246 |
| I-II | 90 (79.6) | 36 (87.8) |  |
| III | 23 (20.4) | 5 (12.2) |  |
| Initial chemotherapy |  |  | 0.907 |
| Yes | 110 (94.8) | 38 (92.7) |  |
| No | 6 (5.2) | 3 (7.3) |  |
| Initial endocrine therapy |  |  | 0.533 |
| Yes | 58 (50.4) | 23 (56.1) |  |
| No | 57 (49.6) | 18 (43.9) |  |
| Initial anti-HER2 target therapy |  |  | 1.000 |
| Yes | 1 (0.9) | 0 (0.0) |  |
| No | 114 (99.1) | 41 (100.0) |  |
| ICWR interval (years) |  |  | 0.323 |
| ≤ 4 | 94 (81.0) | 36 (87.8) |  |
| >4 | 22 (9.0) | 5 (12.2) |  |
| No. of ICWR |  |  | 0.017 |
| 1 | 89 (78.8) | 39 (95.1) |  |
| ≥2 | 24 (21.2) | 2 (4.9) |  |
| Molecular subtype# |  |  | 0.476 |
| Luminal-HER2 negative | 59 (57.3) | 18 (46.2) |  |
| Luminal-HER2 positive | 10 (9.7) | 6 (15.4) |  |
| HER2-enriched | 8 (7.8) | 5 (12.8) |  |
| Triple-negative | 26 (25.2) | 10 (25.6) |  |
| Chemotherapy for ICWR |  |  | 0.481 |
| Yes | 78 (67.2) | 30 (73.2) |  |
| No | 38 (32.8) | 11 (26.8) |  |
| Endocrine therapy for ICWR |  |  | 0.301 |
| Yes | 59 (50.9) | 17 (41.5) |  |
| No | 57 (49.1) | 24 (58.5) |  |
| Anti-HER2 target therapy for ICWR |  |  | 0.067 |
| Yes | 2 (1.7) | 4 (9.8) |  |
| No | 114 (98.3) | 37 (90.2) |  |
| Systemic treatment for ICWR |  |  | 0.088 |
| Yes | 103 (88.8) | 32 (78.0) |  |
| No | 13 (11.2) | 9 (22.0) |  |
| Surgery for ICWR |  |  | 0.233 |
| Yes | 95 (81.9) | 30 (73.2) |  |
| No | 21 (18.1) | 11 (26.8) |  |

*Abbreviations:* HER2 = human epidermal growth factor receptor 2

*For the 17 patients that received neoadjuvant chemotherapy, we used the stage that was higher (clinical or pathological) to reflect the initial tumor burden. # For the 92 patients who had sufficient information for determination of the molecular subtype of ICWR, we used the molecular subtypes of ICWR. For the 94 patients who only had sufficient information for determination of the molecular subtype at initial diagnosis, we used the initial molecular subtypes.
